# A hybrid decision support system with golden cut and bipolar q-ROFSs for evaluating the risk-based strategic priorities of fintech lending for clean energy projects

**DOI:** 10.1186/s40854-022-00406-w

**Published:** 2023-01-07

**Authors:** Qilong Wan, Xiaodong Miao, Chenguang Wang, Hasan Dinçer, Serhat Yüksel

**Affiliations:** 1 School of Economics and Management, Huanghuai University, Zhumadian, 463000 China; 2 School of Economics, University of Chinese Academy of Social Sciences, Beijing, 102488 China; 3 School of Business, Lingnan University, Hong Kong, 999077 China; 4 The School of Business, İstanbul Medipol University, 34817 Beykoz, Istanbul, Turkey

**Keywords:** Fintech lending, Risk management, Clean energy, Fuzzy logic, Decision-making methods

## Abstract

In the last decade, the risk evaluation and the investment decision are among the most prominent issues of efficient project management. Especially, the innovative financial sources could have some specific risk appetite due to the increasing return of investment. Hence, it is important to uncover the risk factors of fintech investments and investigate the possible impacts with an integrated approach to the strategic priorities of fintech lending. Accordingly, this study aims to analyze a unique risk set and the strategic priorities of fintech lending for clean energy projects. The most important contributions to the literature can be listed as to construct an impact-direction map of risk-based strategic priorities for fintech lending in clean energy projects and to measure the possible influences by using a hybrid decision making system with golden cut and bipolar q-rung orthopair fuzzy sets. The extension of multi stepwise weight assessment ratio analysis (M-SWARA) is applied for weighting the risk factors of fintech lending. The extension of elimination and choice translating reality (ELECTRE) is employed for constructing and ranking the risk-based strategic priorities for clean energy projects. In this process, data is obtained with the evaluation of three different decision makers. The main superiority of the proposed model by comparing with the previous models in the literature is that significant improvements are made to the classical SWARA method so that a new technique is created with the name of M-SWARA. Hence, the causality analysis between the criteria can also be performed in this proposed model. The findings demonstrate that security is the most critical risk factor for fintech lending system. Moreover, volume is found as the most critical risk-based strategy for fintech lending. In this context, fintech companies need to take some precautions to effectively manage the security risk. For this purpose, the main risks to information technologies need to be clearly identified. Next, control steps should be put for these risks to be managed properly. Furthermore, it has been determined that the most appropriate strategy to increase the success of the fintech lending system is to increase the number of financiers integrated into the system. Within this framework, the platform should be secure and profitable to persuade financiers.

## Introduction

The costs of clean energy projects are quite expensive compared to fossil fuels. Consequently, investors are becoming less interested in clean energy projects. This situation leads to a decrease in environmentally friendly energy projects. To prevent this problem, clean energy investors must be provided with cost advantages. A fintech lending system can significantly aid in resolving this problem. It is an alternative source of financing for commercial enterprises and consumers in need of capital. Therefore, fintech loan is a next-generation funding system that contributes to the expansion of investments in countries (Chao et al. 2022). Thanks to innovative financial products, investors will have easier access to the necessary financial resources. However, an additional critical issue in this context is the need to effectively analyze ongoing risks (Coşkun and Ibhagui 2022). Otherwise, questions may arise regarding the developed financial products, jeopardizing the projects’ sustainability. In summary, the types of risks in the fintech lending system must be clearly identified. Then, the appropriate measures must be implemented to effectively manage these risks.

The regulatory risk is crucial to this process. Countries can create new regulations for fintech applications (Hai et al. 2022). These new applications may increase the expenses of fintech companies, such as new taxes (Lorenzo and Arroyo 2022). In addition, the new regulations that will be implemented may make it more difficult to enter the sector. This case will diminish the motivation of investors who build the platform toward these practices. Another type of risk in the fintech system is information systems security risk. The fintech platform conducts all transactions over the internet. This increases the risk to security. Moreover, Internet-based attacks by third parties on the fintech platform can cause severe issues (Amarasekara et al. 2022). Because of the security problem in this system, the fintech company’s reputation will suffer significantly. Fintech companies will have difficulty acquiring new customers because of this situation, which will increase their anxiety (Chaudhry et al. 2022). Technology risk should also be considered in this framework (Liu et al. 2022). In the fintech system, where every application is submitted online, a technological glitch may result in customer dissatisfaction (Paramati et al. 2022).

An effective risk management is essential to increase the performance and effectiveness of fintech companies. In this context, fintech companies must take precautions against all types of risk. Meanwhile, each control measure against these risks will increase the companies’ expenses (Thakor et al. 2020). Therefore, it is extremely difficult for fintech companies to take comprehensive precautions against all types of risks. In other words, for fintech companies to become financially efficient, they may need to take some risks. Within this framework, a priority analysis must be conducted for these risk types (Knight and Wojcik 2020). Thanks to this circumstance, we can determine which risks pose the greatest threat to the efficiency of the fintech system. Thus, fintech companies will be better able to implement the risk management procedure, which in turn will not negatively affect the financial performance of these companies.

This study aims to evaluate significant risks and identify the strategic priorities of fintech lending for clean energy projects. Hence, the primary research question is to determine which risks play a more significant role in the effectiveness of the fintech platform. With a comprehensive literature review, this framework defines four distinct risks: regulatory, financial, security, and technological. Therefore, the main hypothesis of this study is that these four risks have a substantial effect on the performance of the fintech platform. This study develops a model to analyze a unique risk set and the strategic priorities of fintech lending for clean energy projects. The risk factors of fintech lending are examined with multi-stepwise weight assessment ratio analysis (M-SWARA) methodology. Furthermore, strategic priorities are evaluated using the elimination and choice translating reality (ELECTRE) method. In this process, the models are integrated with golden cut and bipolar q-rung orthopair fuzzy sets (q-ROFSs). In addition to this concern, all calculations are performed utilizing intuitionistic fuzzy sets (IFSs) and Pythagorean fuzzy sets (PFSs).

This study’s most important contributions to the literature are to construct an impact-direction map of risk-based strategic priorities for fintech lending in clean energy projects and to measure the possible influences using a hybrid decision-making system with golden cut and bipolar q-ROFSs. The analysis enables us to identify more important risks associated with fintech lending. These issues can be extremely useful for defining appropriate risk mitigation strategies for this system. Accordingly, the fintech platform’s effectiveness can be enhanced. Therefore, financial system of the countries can be developed significantly because of a more effective fintech system. Moreover, this situation positively affects the development of environmentally friendly energy projects.

The proposed model has some advantages by comparing with other ones. This study uses enhancements to the classic SWARA system to create a new method called M-SWARA. This new method identifies the impact-relation degrees of the factors. This circumstance provides an important superiority for this model by comparing the previous models in the literature. In other models in that considered SWARA, analytical hierarchy process, or analytical network process, only the weights of criteria can be determined. Due to the operation of the models, the causal relationship could not be identified in these models. Complex and crucial is the issue of evaluating the risk-based strategic priorities of fintech lending for clean energy projects. Thus, the risks can have a substantial impact on one another. For example, regulation risk can impact other risks, such as technological and information security risks. Therefore, to generate appropriate strategies, influenced and influential factors should be defined. Accordingly, M-SWARA methodology is more appropriate for this subject than the other approaches.

Furthermore, golden cut is considered by calculating the degrees in q-ROFSs to increase the appropriateness of the findings. These concerns contribute positively to the originality of the proposed model. Moreover, q-ROFSs consider a wider space by comparing with PFSs and IFSs (Kamacı and Petchimuthu 2022; Lin et al. 2020; 2021). Therefore, more comprehensive evaluations are possible (Li et al. 2020; Garg and Chen 2020; Akçetin and Kamacı 2022). Numerous complexities are involved in evaluating the risk-based strategic priorities of fintech lending for clean energy projects. In this framework, all risks are quite important, so their relative weights of importance are quite close. Due to this issue, identifying more significant risks is extremely challenging. To answer this question, a comprehensive examination must be conducted. Therefore, q-ROFSs are more appropriate for this topic than PFSs and IFSs, since a larger space can be considered during the analysis process (Sahu et al. 2021; Riaz and Fariz 2022). Therefore, these fuzzy sets enable more nuanced assessments.

In addition to q-ROFSs, PFSs and IFSs are used to test the validity of the findings alongside q-ROFSs. The effectiveness of the fintech lending system for clean energy projects is reliant on the identification of more crucial risks. In order to verify the consistency of the analysis results, it is essential to conduct a comparative evaluation using other fuzzy sets. Lastly, the compensation among the factors and the normalization procedure can be avoided by employing the ELECTRE method in the evaluation procedure (Nasution et al. 2020; Biluca et al. 2020). With the aid of this issue, the original data cannot be manipulated, thereby enhancing the applicability of the findings. This is not the case for other comparable techniques described in the literature, such as technique for order preference by similarity to ideal solution (TOPSIS) (Vojinović et al. 2022). In addition, when employing bipolar fuzzy sets, both negative and positive sets can be considered to obtain more comprehensive data by comparing with other fuzzy sets (Akram and Arshad 2020; Riaz and Tehrim, 2020; Shumaiza et al., 2019). Thus, it is possible to achieve more precise results (Akram and Al-Kenani 2020; Akram et al. 2019; Akram and Arshad 2019; Alghamdi et al. 2018).

In Sect. 2, the literature on risks in fintech lending is explained. Methods are described in detail in Sect. 3. The model’s results are presented in Sect. 4. In Sects. 5 and 6, conclusions and discussions are presented.

## Literature for risks in fintech lending

This section contains a literature review on the risks associated with fintech lending systems. In evaluating the literature, the two most recent studies are considered. The studies are selected from the social science citation index-indexed journals. A unique paragraph is created for each type of the risk. Information systems security risk is an important type of risk that has an impact on the effectiveness of the fintech lending process. The fintech platform conducts all operations via the internet, which increases the platform’s information security risks (Hwang et al. 2021). As a result of these risks not being effectively managed, serious system problems may arise (Miyauchi 2021). Since the fintech company is responsible for the platform’s security, it is also responsible for any security-related issues that may arise (Hussain et al. 2021). Therefore, the problems that will arise due to the lack of security will result in significant losses for the fintech company (Maiti and Ghosh 2021). Within this framework, fintech firms must define all risks in detail (Meng et al. 2021). The next step is identifying and implementing the necessary control measures for these risks. Najib et al. (2021) investigated the influence of fintech systems on economic growth. They underlined that security conditions should be satisfied. Le (2021) evaluated the fintech system after COVID-19 period and identified that necessary actions should be taken to address security issues.

Technology risk is another important type of risk for the efficient development of fintech lending processes. The fintech lending process is conducted entirely online. Therefore, in order for this system to function properly, a robust technological infrastructure is required (Yusuf 2021). Without adequate technology, the fintech platform will be plagued by persistent bugs (Wang et al. 2022). Consequently, customer discontent will increase. This will result in the loss of customers for the fintech company (Chaudhry et al. 2022). Consequently, fintech firms must prioritize technological investment (Coffie et al. 2021). In this manner, the technological risk on the platform will be reduced to an absolute minimum. Setiawan et al. (2021) examined the Indonesian fintech system. For the effectiveness of the fintech lending system, they emphasized the need for necessary technological development. Sheng (2021) studied the performance of the fintech system for different country groups and determined the increase in customer dissatisfaction with technological problems in the system.

Financial risk is another type of risk that must be considered to improve the performance of the fintech lending system. Financial risk is the possibility that a fintech company will be unable to meet its obligations due to insufficient assets (Banna et al. 2021). Companies with funds on the fintech platform and those in need of funds are brought together on the internet (Zhao et al. 2022). In this context, the associated fund is made available as a loan to customers (Sakarya and Aksu 2021). The creditworthiness of the customers must be high (Al Janabi 2021). If the loans given to individuals with low creditworthiness are not repaid, the fintech company is exposed to financial risk. Liu (2021) examined methods for enhancing the efficacy of the fintech system. Therefore, fintech companies should conduct an effective customer credibility analysis. Katsiampa et al. (2022) evaluated the fintech system in China and determined that financial risks must be effectively managed to enhance the system.

Regulation risk should also be considered for a fintech lending system to be effective. This risk refers to the possibility that a change in laws and regulations will significantly affect the fintech company. Countries are able to enact new rules for the fintech system (Ebrahim et al. 2021). These new applications may also increase the expenses of fintech firms. In addition, new regulations may discourage investors from entering the market (Xu et al. 2021). For instance, the additional taxes imposed on fintech applications can substantially increase investor costs (Wojcik 2021). This situation endangers the sustainability of this system because it will negatively impact profitability (Omarova 2021). Omarini (2021) focused on the critical issues for enhancing the fintech system’s performance. To achieve this objective, fintech companies should primarily consider regulatory risks. Huibers (2021) examined the fintech credit system in the Netherland and pointed out that necessary actions should be taken regarding the regulation risks for the success of this system.

The results of the literature review indicate that the fintech lending system is subject to a variety of risks. Therefore, businesses must take precautions for each type of risk. However, the measures taken for each type of risk incur additional business expenses. Therefore, fintech companies cannot take comprehensive precautions against all potential threats. In this context, it is necessary to establish risk priorities for the fintech system to identify the most important issues. However, only a few studies in the literature have focused on the analysis with respect to the risks in the fintech lending system. Accordingly, the present study aims to assess significant risks and determine the strategic priorities of fintech lending for clean energy projects. By focusing on this topic, this study is believed to contribute to the existing body of knowledge.

## Methodology

Bipolar q-ROFSs, SWARA and ELECTRE are detailed in this part.

### Bipolar q-ROFSs with golden cut

Atanassov (1999) generated IFSs with membership and non-membership (MGS and NGS) degrees ($\mu_{I} , n_{I} )$ in Eq. (1).

1 $$I = \left\{ {\vartheta ,\mu_{I} \left( \vartheta \right),n_{I} \left( \vartheta \right)/\vartheta \varepsilon U} \right\}$$

Equation (2) includes the requirement.

2 $$0 \le \mu_{I} \left( \vartheta \right) + n_{I} \left( \vartheta \right) \le 1$$

PFSs are introduced by Yager (2013) with degrees ($\mu_{p} , n_{p} )$ as in Eq. (3).

3 $$P = \left\{ {\vartheta ,\mu_{P} \left( \vartheta \right),n_{P} \left( \vartheta \right)/\vartheta \varepsilon U} \right\}$$

Equation (4) states the requirement.

4 $$0 \le \left( {\mu_{P} \left( \vartheta \right)} \right)^{2} + \left( {n_{P} \left( \vartheta \right)} \right)^{2} \le 1$$

Also, q-ROFSs are developed by Yager (2016) with the extension of PFSs and IFSs as in Eq. (5).

5 $$Q = \left\{ {\vartheta ,\mu_{Q} \left( \vartheta \right),n_{Q} \left( \vartheta \right)/\vartheta \varepsilon U} \right\}$$

The requirement is given in Eq. (6).

6 $$0 \le \left( {\mu_{Q} \left( \vartheta \right)} \right)^{q} + \left( {n_{Q} \left( \vartheta \right)} \right)^{q} \le 1 , q \ge 1$$

Zhang (1994) generated bipolar fuzzy sets to better reflect uncertainties. Equation (7) describes them, where $\mu_{B}^{ + }$ states the satisfaction degree and satisfaction of the same element is shown by $\mu_{B}^{ - }$.

7 $$B = \left\{ {\vartheta , \mu_{B}^{ + } \left( \vartheta \right),\mu_{B}^{ - } \left( \vartheta \right)/\vartheta \varepsilon U} \right\}$$

**Fig. 1:**
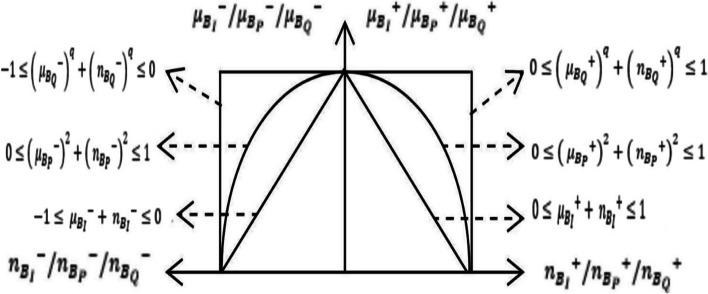
Degrees of bipolar IFS, PFS, and q-ROFSs

Bipolar fuzzy sets can be adopted to IFSs, PFSs and q-ROFSs as in Eqs. (8–13).

8 $$B_{I} = \left\{ {\vartheta , \mu_{{B_{I} }}^{ + } \left( \vartheta \right),n_{{B_{I} }}^{ + } \left( \vartheta \right),\mu_{{B_{I} }}^{ - } \left( \vartheta \right),n_{{B_{I} }}^{ - } \left( \vartheta \right)/\vartheta \varepsilon U} \right\}$$

9 $$B_{P} = \left\{ {\vartheta , \mu_{{B_{P} }}^{ + } \left( \vartheta \right),n_{{B_{P} }}^{ + } \left( \vartheta \right),\mu_{{B_{P} }}^{ - } \left( \vartheta \right),n_{{B_{P} }}^{ - } \left( \vartheta \right)/\vartheta \varepsilon U} \right\}$$

10 $$B_{Q} = \left\{ {\vartheta , \mu_{{B_{Q} }}^{ + } \left( \vartheta \right),n_{{B_{Q} }}^{ + } \left( \vartheta \right),\mu_{{B_{Q} }}^{ - } \left( \vartheta \right),n_{{B_{Q} }}^{ - } \left( \vartheta \right)/\vartheta \varepsilon U} \right\}$$

11 $$0 \le \left( { \mu_{{B_{I} }}^{ + } \left( \vartheta \right)} \right)^{{}} + \left( { n_{{B_{I} }}^{ + } \left( \vartheta \right)} \right)^{{}} \le 1,\; - 1 \le \left( { \mu_{{B_{I} }}^{ - } \left( \vartheta \right)} \right)^{{}} + \left( { n_{{B_{I} }}^{ - } \left( \vartheta \right)} \right)^{{}} \le 0$$

12 $$0 \le \left( { \mu_{{B_{P} }}^{ + } \left( \vartheta \right)} \right)^{2} + \left( { n_{{B_{P} }}^{ + } \left( \vartheta \right)} \right)^{2} \le 1,\;0 \le \left( { \mu_{{B_{P} }}^{ - } \left( \vartheta \right)} \right)^{2} + \left( { n_{{B_{P} }}^{ - } \left( \vartheta \right)} \right)^{2} \le 1$$

13 $$0 \le \left( { \mu_{{B_{Q} }}^{ + } \left( \vartheta \right)} \right)^{q} + \left( { n_{{B_{Q} }}^{ + } \left( \vartheta \right)} \right)^{q} \le 1,\; - 1 \le \left( { \mu_{{B_{Q} }}^{ - } \left( \vartheta \right)} \right)^{q} + \left( { n_{{B_{Q} }}^{ - } \left( \vartheta \right)} \right)^{q} \le 0$$

where $\mu_{{B_{I} }}^{ + } ,\mu_{{B_{P} }}^{ + } ,\mu_{{B_{Q} }}^{ + } , n_{{B_{I} }}^{ + } ,n_{{B_{P} }}^{ + } ,n_{{B_{Q} }}^{ + } :U \to \left[ {0,1} \right]$ and define the positive member and non-membership degrees. $\mu_{{B_{I} }}^{ - } ,\mu_{{B_{P} }}^{ - } ,\mu_{{B_{Q} }}^{ - } , n_{{B_{I} }}^{ - } ,n_{{B_{P} }}^{ - } ,n_{{B_{Q} }}^{ - } :U \to \left[ { - 1,0} \right]$ and are the negative member and non-membership degrees for bipolar IFS, PFS, and q-ROFS respectively. For bipolar q-ROFS, $q$ is defined as odd number. The details are illustrated in Fig. 1.

Operations are given in Eqs. (14–17).

$$B_{Q1} = \left\{ {\vartheta , \mu_{{B_{Q1} }}^{ + } \left( \vartheta \right),n_{{B_{Q1} }}^{ + } \left( \vartheta \right),\mu_{{B_{Q1} }}^{ - } \left( \vartheta \right),n_{{B_{Q1} }}^{ - } \left( \vartheta \right)/\vartheta \varepsilon U} \right\}$$ and

$$B_{Q2} = \left\{ {\vartheta , \mu_{{B_{Q2} }}^{ + } \left( \vartheta \right),n_{{B_{Q2} }}^{ + } \left( \vartheta \right),\mu_{{B_{Q2} }}^{ - } \left( \vartheta \right),n_{{B_{Q2} }}^{ - } \left( \vartheta \right)/\vartheta \varepsilon U} \right\}$$

14 $$\begin{aligned} B_{Q1} \oplus B_{Q2} & = \left( {\left( {\left( {\mu_{{B_{Q1} }}^{ + } } \right)^{q} + \left( {\mu_{{B_{Q2} }}^{ + } } \right)^{q} - \left( {\mu_{{B_{Q1} }}^{ + } } \right)^{q} .\left( {\mu_{{B_{Q2} }}^{ + } } \right)^{q} } \right)^{\frac{1}{q}} , \left( {n_{{B_{Q1} }}^{ + } . n_{{B_{Q2} }}^{ + } } \right), - \left( {\mu_{{B_{Q1} }}^{ - } . \mu_{{B_{Q2} }}^{ - } } \right),} \right. \\ & \left. {\quad - \left( {\left( {n_{{B_{Q1} }}^{ - } } \right)^{q} + \left( {n_{{B_{Q2} }}^{ - } } \right)^{q} - \left( {n_{{B_{Q1} }}^{ - } } \right)^{q} .\left( {n_{{B_{Q2} }}^{ - } } \right)^{q} } \right)^{\frac{1}{q}} } \right) \\ \end{aligned}$$

15 $$\begin{aligned} B_{Q1} \otimes B_{Q2} & = \left( {\left( {\mu_{{B_{Q1} }}^{ + } .\mu_{{B_{Q2} }}^{ + } } \right), \left( {\left( {n_{{B_{Q1} }}^{ + } } \right)^{q} + \left( {n_{{B_{Q2} }}^{ + } } \right)^{q} - \left( {n_{{B_{Q1} }}^{ + } } \right)^{q} .\left( {n_{{B_{Q2} }}^{ + } } \right)^{q} } \right)^{\frac{1}{q}} ,} \right. \\ & \left. {\quad - \left( {\left( {\mu_{{B_{Q1} }}^{ - } } \right)^{q} + \left( {\mu_{{B_{Q2} }}^{ - } } \right)^{q} - \left( {\mu_{{B_{Q1} }}^{ - } } \right)^{q} .\left( {\mu_{{B_{Q2} }}^{ - } } \right)^{q} } \right)^{\frac{1}{q}} , - \left( {n_{{B_{Q1} }}^{ - } . n_{{B_{Q2} }}^{ - } } \right)} \right) \\ \end{aligned}$$

16 $$\lambda B_{Q1} = \left( {\left( {1 - \left( {1 - \left( {\mu_{{B_{Q1} }}^{ + } } \right)^{q} } \right)^{\lambda } } \right)^{1/q} , \left( {n_{{B_{Q1} }}^{ + } } \right)^{\lambda } , - \left( { - \mu_{{B_{Q1} }}^{ - } } \right)^{\lambda } , - \left( {1 - \left( {1 - \left( { - n_{{B_{Q1} }}^{ - } } \right)^{q} } \right)^{\lambda } } \right)^{1/q} } \right),\lambda > 0 ^{{}}$$

17 $$B_{Q1}^{\lambda } = \left( {\left( {\mu_{{B_{Q1} }}^{ + } } \right)^{\lambda } , \left( {1 - \left( {1 - \left( {n_{{B_{Q1} }}^{ + } } \right)^{q} } \right)^{\lambda } } \right)^{1/q} , - \left( {1 - \left( {1 - \left( { - \mu_{{B_{Q1} }}^{ - } } \right)^{q} } \right)^{\lambda } } \right)^{\frac{1}{q}} , - \left( { - n_{{B_{Q1} }}^{ - } } \right)^{\lambda } } \right),\lambda > 0 ^{{}}$$

Equations (18–20) are used for defuzzification.

18 $$S\left( \vartheta \right)_{{B_{I} }} = \left( {\left( {\mu_{{B_{I} }}^{ + } \left( \vartheta \right)} \right)^{{}} - \left( {n_{{B_{I} }}^{ + } \left( \vartheta \right)} \right)^{{}} } \right) - \left( {\left( {\mu_{{B_{I} }}^{ - } \left( \vartheta \right)} \right)^{{}} - \left( {n_{{B_{I} }}^{ - } \left( \vartheta \right)} \right)^{{}} } \right)$$

19 $$S\left( \vartheta \right)_{{B_{P} }} = \left( {\left( {\mu_{{B_{P} }}^{ + } \left( \vartheta \right)} \right)^{2} - \left( {n_{{B_{P} }}^{ + } \left( \vartheta \right)} \right)^{2} } \right) + \left( {\left( {\mu_{{B_{P} }}^{ - } \left( \vartheta \right)} \right)^{2} - \left( {n_{{B_{P} }}^{ - } \left( \vartheta \right)} \right)^{2} } \right)$$

20 $$S\left( \vartheta \right)_{{B_{Q} }} = \left( {\left( {\mu_{{B_{Q} }}^{ + } \left( \vartheta \right)} \right)^{q} - \left( {n_{{B_{Q} }}^{ + } \left( \vartheta \right)} \right)^{q} } \right) - \left( {\left( {\mu_{{B_{Q} }}^{ - } \left( \vartheta \right)} \right)^{q} - \left( {n_{{B_{Q} }}^{ - } \left( \vartheta \right)} \right)^{q} } \right)$$

This study considers golden cut ($\varphi )$ to calculate the degrees. Equation (21) details this by denoting large and small quantities by *a* and *b* (Hu et al. 2020).

21 $$\varphi = \frac{a}{b}$$

Equation (22) includes the arithmetical illustration.

22 $$\varphi = \frac{1 + \sqrt 5 }{2} = 1.618 \ldots$$

Equation (23) states the degrees ($\mu_{{G_{{B_{Q} }} }}$, $n_{{G_{{B_{Q} }} }}$).

23 $$\varphi = \frac{{\mu_{{G_{{B_{Q} }} }} }}{{n_{{G_{{B_{Q} }} }} }}$$

Equations (24)-(26) include the adopted on golden cut to bipolar fuzzy sets.

24 $$G_{{B_{Q} }} = \left\{ {\vartheta , \mu_{{G_{{B_{Q} }} }}^{ + } \left( \vartheta \right),n_{{G_{{B_{Q} }} }}^{ + } \left( \vartheta \right),\mu_{{G_{{B_{Q} }} }}^{ - } \left( \vartheta \right),n_{{G_{{B_{Q} }} }}^{ - } \left( \vartheta \right)/\vartheta \varepsilon U} \right\}$$

25 $$0 \le \left( { \mu_{{G_{{B_{Q} }} }}^{ + } \left( \vartheta \right)} \right)^{q} + \left( { n_{{G_{{B_{Q} }} }}^{ + } \left( \vartheta \right)} \right)^{q} \le 1,\; - 1 \le \left( { \mu_{{G_{{B_{Q} }} }}^{ - } \left( \vartheta \right)} \right)^{q} + \left( { n_{{G_{{B_{Q} }} }}^{ - } \left( \vartheta \right)} \right)^{q} \le 0$$

26 $$0 \le \left( { \mu_{{G_{{B_{Q} }} }}^{ + } \left( \vartheta \right)} \right)^{2q} + \left( { n_{{G_{{B_{Q} }} }}^{ + } \left( \vartheta \right)} \right)^{2q} \le 1,\;0 \le \left( { \mu_{{G_{{B_{Q} }} }}^{ - } \left( \vartheta \right)} \right)^{2q} + \left( { n_{{G_{{B_{Q} }} }}^{ - } \left( \vartheta \right)} \right)^{2q} \le 1\; q \ge 1$$

### M-SWARA method with bipolar q-ROFSs

Keršuliene et al. (2010) developed SWARA to compute the weights of the factors. In this method, the expert team can select the priorities. Equation (27) provides details about the relationship matrix.

27 $$Q_{k} = \left[ {\begin{array}{*{20}c} 0 & {Q_{12} } & \cdots & {} & \cdots & {Q_{1n} } \\ {Q_{21} } & 0 & \cdots & {} & \cdots & {Q_{2n} } \\ \vdots & \vdots & \ddots & {} & \cdots & \cdots \\ \vdots & \vdots & \vdots & {} & \ddots & \vdots \\ {Q_{n1} } & {Q_{n2} } & \cdots & {} & \cdots & 0 \\ \end{array} } \right]$$

Score functions and bipolar fuzzy sets are created. Equations (28–30) explain the values of comparative significance ratio ($$s_{j} )$$, coefficient ($$k_{j} )$$, recomputed weight ($$q_{j} )$$, and weight ($$w_{j}$$).

28 $$k_{j} = \left\{ {\begin{array}{*{20}c} {1 j = 1} \\ {s_{j} + 1 j > 1} \\ \end{array} } \right.$$

29 $$q_{j} = \left\{ {\begin{array}{*{20}c} {1 j = 1} \\ {\frac{{q_{j - 1} }}{{k_{j} }} j > 1} \\ \end{array} } \right.$$

$$If s_{j - 1} = s_{j} , q_{j - 1} = q_{j} ;\;If s_{j} = 0, k_{j - 1} = k_{j}$$

30 $$w_{j} = \frac{{q_{j} }}{{\mathop \sum \nolimits_{k = 1}^{n} q_{k} }}$$

Stable matrix is constructed by limiting and taking transpose of the matrix with the power of 2t + 1.

### ELECTRE with bipolar q-ROFSs

Benayoun et al. (1966) developed ELECTRE by considering binary superiority comparisons to rank the items. Equation (31) includes the decision matrix.

31 $$X_{k} = \left[ {\begin{array}{*{20}c} 0 & {X_{12} } & \cdots & {} & \cdots & {X_{1m} } \\ {X_{21} } & 0 & \cdots & {} & \cdots & {X_{2m} } \\ \vdots & \vdots & \ddots & {} & \cdots & \cdots \\ \vdots & \vdots & \vdots & {} & \ddots & \vdots \\ {X_{n1} } & {X_{n2} } & \cdots & {} & \cdots & 0 \\ \end{array} } \right]$$

This matrix is normalized by Eq. (32).

32 $$r_{ij} = \frac{{X_{ij} }}{{\sqrt {\mathop \sum \nolimits_{i = 1}^{m} X_{ij}^{2} } }}.$$

In this framework, if $X_{ij}$ is equal to “0” for all i and j, then $r_{ij}$ will be undefined because of the value of “0/0”. The values are weighted with Eq. (33).

33 $$v_{ij} = w_{ij} \times r_{ij}$$

Equations (34–39) explain the calculation of concordance and discordance (*C* and *D)* interval matrices.

34 $$C = \left[ {\begin{array}{*{20}c} - & {c_{12} } & \cdots & {} & \cdots & {c_{1n} } \\ {c_{21} } & - & \cdots & {} & \cdots & {c_{2n} } \\ \vdots & \vdots & \ddots & {} & \cdots & \cdots \\ \vdots & \vdots & \vdots & {} & \ddots & \vdots \\ {c_{n1} } & {c_{n2} } & \cdots & {} & \cdots & - \\ \end{array} } \right]$$

35 $$D = \left[ {\begin{array}{*{20}c} - & {d_{12} } & \cdots & {} & \cdots & {d_{1n} } \\ {d_{21} } & - & \cdots & {} & \cdots & {d_{2n} } \\ \vdots & \vdots & \ddots & {} & \cdots & \cdots \\ \vdots & \vdots & \vdots & {} & \ddots & \vdots \\ {d_{n1} } & {d_{n2} } & \cdots & {} & \cdots & - \\ \end{array} } \right]$$

36 $$c_{ab} = \left\{ {j{|}v_{aj} \ge v_{bj} } \right\}$$

37 $$d_{ab} = \left\{ {j{|}v_{aj} < v_{bj} } \right\}$$

38 $$c_{ab} = \mathop \sum \limits_{{j \in c_{ab} }} w_{j}$$

39 $$d_{ab} = \frac{{max_{{j \in d_{ab} }} \left| {v_{aj} - v_{bj} } \right|}}{{max_{j} \left| {v_{mj} - v_{nj} } \right|}}$$

Equations (40–47) include the creation of the concordance *E*, discordance *F* and aggregated *G* index matrixes.

40 $$E = \left[ {\begin{array}{*{20}c} - & {e_{12} } & \cdots & {} & \cdots & {e_{1n} } \\ {e_{21} } & - & \cdots & {} & \cdots & {e_{2n} } \\ \vdots & \vdots & \ddots & {} & \cdots & \cdots \\ \vdots & \vdots & \vdots & {} & \ddots & \vdots \\ {e_{n1} } & {e_{n2} } & \cdots & {} & \cdots & - \\ \end{array} } \right]$$

41 $$F = \left[ {\begin{array}{*{20}c} - & {f_{12} } & \cdots & {} & \cdots & {f_{1n} } \\ {f_{21} } & - & \cdots & {} & \cdots & {f_{2n} } \\ \vdots & \vdots & \ddots & {} & \cdots & \cdots \\ \vdots & \vdots & \vdots & {} & \ddots & \vdots \\ {f_{n1} } & {f_{n2} } & \cdots & {} & \cdots & - \\ \end{array} } \right]$$

42 $$G = \left[ {\begin{array}{*{20}c} - & {g_{12} } & \cdots & {} & \cdots & {g_{1n} } \\ {g_{21} } & - & \cdots & {} & \cdots & {g_{2n} } \\ \vdots & \vdots & \ddots & {} & \cdots & \cdots \\ \vdots & \vdots & \vdots & {} & \ddots & \vdots \\ {g_{n1} } & {g_{n2} } & \cdots & {} & \cdots & - \\ \end{array} } \right]$$

43 $$\left\{ {\begin{array}{*{20}c} {e_{ab} = 1 if c_{ab} \ge \overline{c}} \\ {e_{ab} = 0 if c_{ab} < \overline{c}} \\ \end{array} } \right.$$

44 $$\overline{c} = \mathop \sum \limits_{a = 1}^{n} \mathop \sum \limits_{b}^{n} c_{ab} /n\left( {n - 1} \right)$$

45 $$\left\{ {\begin{array}{*{20}c} {f_{ab} = 1 if d_{ab} \le \overline{d}} \\ {f_{ab} = 0 if d_{ab} > \overline{d}} \\ \end{array} } \right.$$

46 $$\overline{d} = \mathop \sum \limits_{a = 1}^{n} \mathop \sum \limits_{b}^{n} d_{ab} /n\left( {n - 1} \right)$$

47 $$g_{ab} = e_{ab} \times f_{ab}$$

In this scope, $e_{ab}$, $f_{ab}$, and $g_{ab}$ refer to the sets of concordance, discordance, and aggregated index matrixes, respectively. Also, the net superior $c_{a}$, inferior $d_{a}$, and overall $o_{a}$ values are computed by the following equations:

48 $$c_{a} = \mathop \sum \limits_{b = 1}^{n} c_{ab} - \mathop \sum \limits_{b = 1}^{n} c_{ba}$$

49 $$d_{a} = \mathop \sum \limits_{b = 1}^{n} d_{ab} - \mathop \sum \limits_{b = 1}^{n} d_{ba}$$

50 $$o_{a} = c_{a} - d_{a}$$

## Analysis

**Fig. 2:**
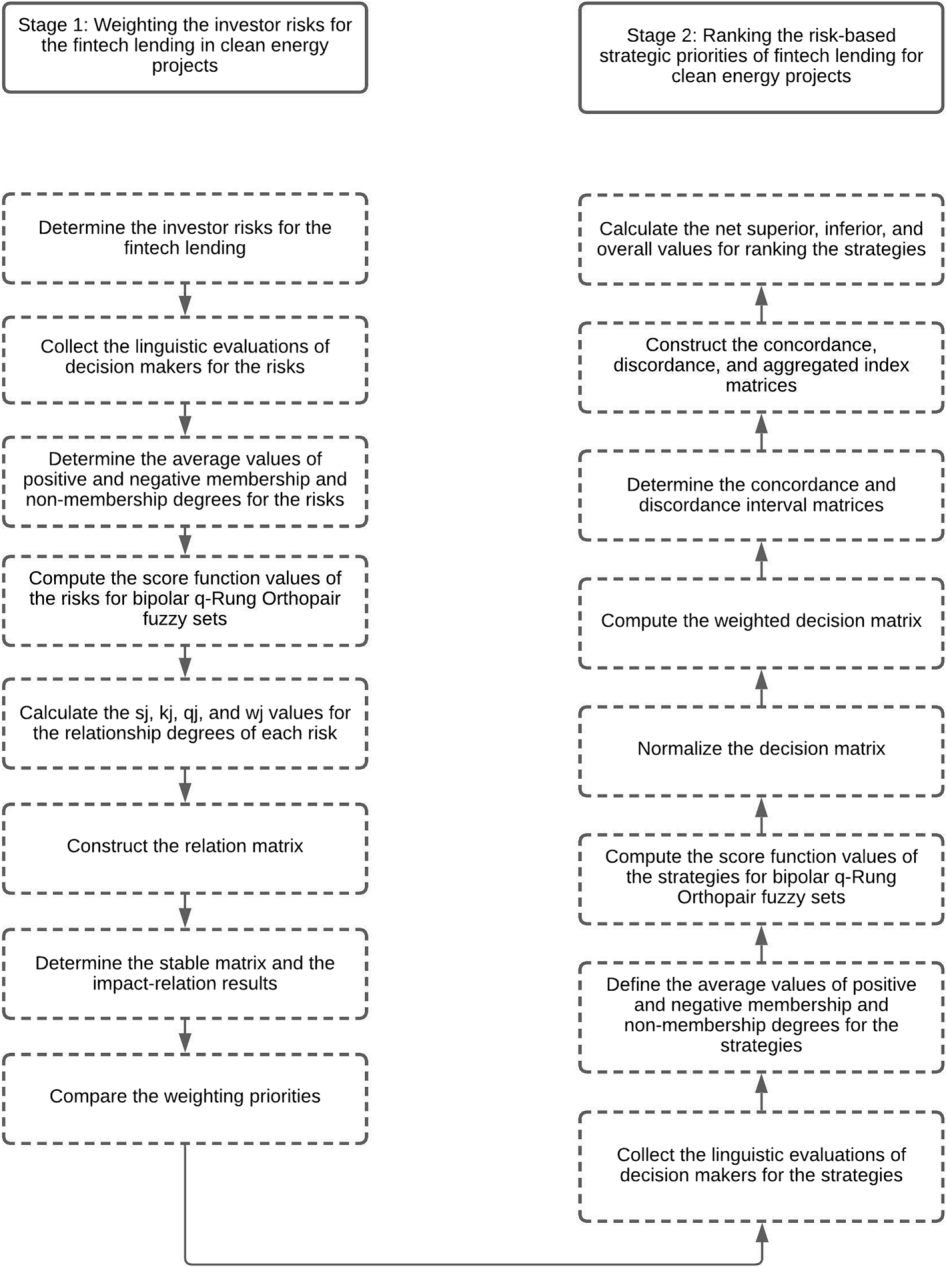
Proposed model

Clean energy projects have high initial cost that is accepted as the main drawback of these projects. Thus, innovative financial technology products play a crucial role in these endeavors. Consequently, the purpose of this study is to analyze a distinct risk set and the strategic priorities of fintech lending for clean energy projects. In order to analyze a unique risk set and the strategic priorities of fintech lending for clean energy projects, a model is developed in this study. This proposed model consists primarily of two distinct phases. First, the investor risks associated with fintech lending for clean energy projects are quantified. Second, the risk-based strategic priorities of fintech lending for clean energy projects are ranked. All phases are explained in Fig. 2.

*Stage 1* Weighting the investors’ risks for the fintech lending in clean energy projects.

*Step 1* Determine the investor risks for the fintech lending.

The investor risks associated with fintech lending are outlined in Table 1. These factors represent the dangers for businesses that develop fintech lending platforms to attract clean energy investors.

**Table 1:** Selected investor risks for the fintech lending

Risks	Details of the risks	References
Regulation (RLN)	the legal rules in the country, making rapid changes in legal regulations	Huibers (2021)
Financial (LDT)	volatility in the currency, liquidity risks, financial performance risks	
Security (STY)	security related to the web site, taking precautions for the hacking attacks	Setiawan et al. (2021)
Technological (TGL)	the company’s technological inadequacy, the lack of effective monitoring of current technological developments	Najib et al. (2021)

Regulation risk plays a significant role in the fintech lending system. It is defined as regulatory changes that negatively impact the fintech lending system. Financial risk indicates that fintech lending companies lack the liquid assets necessary to meet their obligations. Security risk refers to the possibility of unauthorized access to the information technology (IT) systems of the fintech platform. Technological risk provides information about the disruption of processes caused by a lack of technological expertise during research and development by fintech companies. Table 2 contains the scales and degrees utilized in the calculations, where PDG and NDG stand for positive and negative degrees, respectively.

**Table 2:** Scale and degrees

Scales		PDG		NDG	
for Risks	for Strategies	MGS	NGS	MGS	NGS
No (n)	Weakest (w)	.40	.25	− .60	− .37
Somewhat (s)	Poor (p)	.45	.28	− .55	− .34
Medium (m)	Fair (f)	.50	.31	− .50	− .31
High (h)	Good (g)	.55	.34	− .45	− .28
Very high (vh)	Best (b)	.60	.37	− .40	− .25

*Step 2* Collect the linguistic evaluations.

Table 3 displays evaluations of risks. For this purpose, an expert team is created with three different decision-makers. This group has a minimum of 24 years of experience in financial technology. They serve as senior executives for various fintech companies. They have worked in various departments of financial technology companies until now. Thus, they were able to gain a variety of experiences with financial technology applications. Therefore, these experts have sufficient knowledge to make an assessment on this subject.

**Table 3:** Evaluations for risks

	RLN		LDT		STY		TGL	
	PDG	NDG	PDG	NDG	PDG	NDG	PDG	NDG
*Decision maker 1*								
RLN			M	M	M	N	S	H
LDT	H	H			VH	H	M	M
STY	H	VH	H	M			S	M
TGL	VH	M	H	N	VH	N		
*Decision maker 2*								
RLN			H	M	M	N	VH	VH
LDT	H	H			VH	M	M	M
STY	VH	VH	H	VH			VH	M
TGL	VH	M	H	N	VH	N		
*Decision maker 3*								
RLN			M	S	M	S	S	H
LDT	S	S			VH	M	M	N
STY	S	VH	H	M			S	M
TGL	H	M	H	N	H	N		

*Step 3* Determine the average values of positive and negative membership and non-membership degrees for the risks.

Average values are indicated in Table 4.

**Table 4:** Average values for the risks

	RLN				LDT				STY				TGL			
	PDG		NDG		PDG		NDG		PDG		NDG		PDG		NDG	
	μ	n	μ	n	μ	n	μ	n	μ	n	μ	n	μ	n	μ	n
RLN					.52	.32	− .52	− .32	.50	.31	− .58	− .36	.50	.31	− .43	− .27
LDT	.52	.32	− .48	− .30					.60	.37	− .48	− .30	.50	.31	− .53	− .33
STY	.53	.33	− .40	− .25	.55	.34	− .47	− .29					.50	.31	− .50	− .31
TGL	.58	.36	− .50	− .31	.55	.34	− .60	− .37	.58	.36	− .60	− .37				

*Step 4* Compute the score function values.

The values of score functions are calculated in Table 5.

**Table 5:** Score function values of the risks

	RLN	LDT	STY	TGL
RLN	.000	.211	.247	.158
LDT	.192	.000	.251	.211
STY	.165	.205	.000	.191
TGL	.247	.292	.317	.000

*Step 5* Compute s_j_, k_j_, q_j_, and w_j_ values.

SWARA approach is extended in this study by the name of M-SWARA. Within this context, some improvements are made, such as computing s_j_, k_j_, q_j_, and w_j_ values with the help of Eqs. (28–30). In this framework, $k_{j}$ refers to the coefficient value, $q_{j}$ shows the recalculated weight, $s_{j}$ indicates the comparative importance rate and $w_{j}$ represents the weights of the criteria. These values are computed to define the criteria relationship degrees. The specifics of these values are detailed in Table 6.

**Table 6:** sj, kj, qj, and wj values for the relationship degrees of each risk

RLN	s_j_	k_j_	q_j_	W_j_	LDT	s_j_	k_j_	q_j_	w_j_
STY	.247	1.000	1.000	.394	STY	.251	1.000	1.000	.397
LDT	.211	1.211	.826	.325	TGL	.211	1.211	.826	.328
TGL	.158	1.158	.713	.281	RLN	.192	1.192	.693	.275

STY	s_j_	k_j_	q_j_	W_j_	TGL	s_j_	k_j_	q_j_	w_j_
LDT	.205	1.000	1.000	.391	STY	.317	1.000	1.000	.418
TGL	.191	1.191	.840	.328	LDT	.292	1.292	.774	.323
RLN	.165	1.165	.721	.282	RLN	.247	1.247	.621	.259

*Step 6* Construct the relation matrix.

Relation matrix is generated in Table 7.

**Table 7:** Relation matrix

	RLN	LDT	STY	TGL
RLN		.325	.394	.281
LDT	.275		.397	.328
STY	.282	.391		.328
TGL	.259	.323	.418	

*Step 7* Determine the stable matrix.

Stable matrix is developed for computing the weights of the items in Table 8.

**Table 8:** Stable matrix

	RLN	LDT	STY	TGL
RLN	.214	.214	.214	.214
LDT	.259	.259	.259	.259
STY	.287	.287	.287	.287
TGL	.239	.239	.239	.239

*Step 8* Compare weighting priorities.

**Fig. 3:**
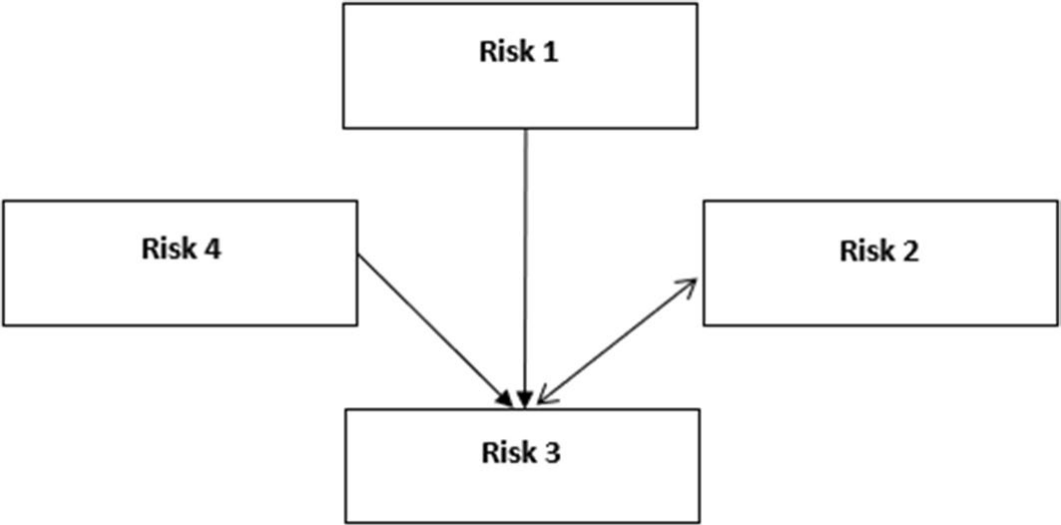
Causal degrees for the risks

The causal relationship among the risk factors is identified in Fig. 3.

Security is affected by three other risk factors. Additionally, security influences the financial realm. This calculation is also performed using IFSs and PFSs. Table 9 provides a summary of all conclusions.

**Table 9:** Weighting priorities for the risks

	Bipolar IFSs	Bipolar PFSs	Bipolar q-ROFSs
RLN	4	4	4
LDT	2	2	2
STY	1	1	1
TGL	3	3	3

**Fig. 4:**
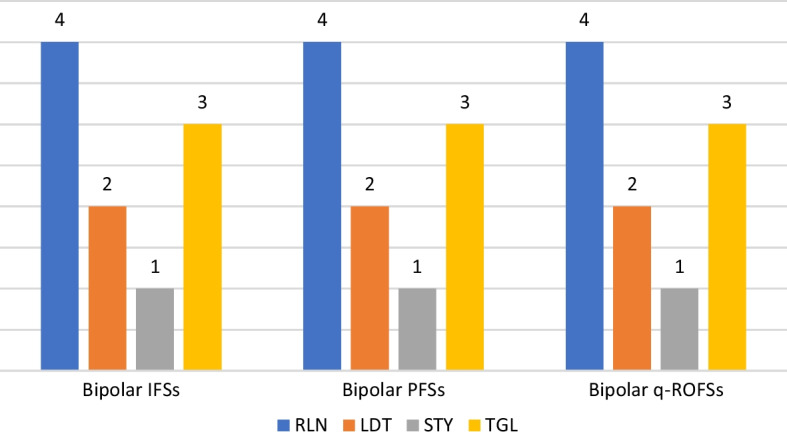
Graphical representations of the weighting priorities

The most important risk factor for a fintech lending system is security. Moreover, financial has the second-best rank for this issue. Financial and technological risks carry less weight. Figure 4 depicts the specifics of the risk weighting priorities.

*Stage 2* Ranking the risk-based strategic priorities of fintech lending for clean energy projects.

*Step 9* Collect the linguistic evaluations for the strategies.

In the second stage of the proposed model, the risk-based strategic priorities of fintech lending for clean energy projects are ranked based on their strategic importance. Table 10 displays a selection of strategic priorities based on risk.

**Table 10:** Selected risk-based strategic priorities of fintech lending for clean energy projects

Strategies	References
Cost	Hwang et al. (2021)
Time	Yusuf (2021)
Volume	Omara (2021)
Information	Banna et al. (2021)

Cost refers to the decrease in operational expenses as a result of fewer personnel and physical inputs. In addition, time includes the short-term matching of fund suppliers with fund demanders. Furthermore, volume means providing a wide set of finance providers for the different projects. The information presents the project data and the rating of investors clearly. Table 11 depicts evaluations of strategies.

**Table 11:** Evaluations for strategies

	RLN		LDT		STY		TGL	
	PDG	NDG	PDG	NDG	PDG	NDG	PDG	NDG
*Decision maker 1*								
Cost	B	W	F	P	B	G	P	G
Time	P	F	F	P	G	B	G	G
Volume	B	B	G	W	P	F	B	W
Information	F	W	P	B	P	G	G	B
*Decision maker 2*								
Cost	G	P	F	P	B	G	P	G
Time	F	P	F	P	B	B	B	G
Volume	G	G	G	W	F	F	B	W
Information	G	F	F	P	P	F	G	F
*Decision maker 3*								
Cost	G	P	F	P	B	G	F	G
Time	F	P	F	W	G	B	G	G
Volume	B	B	G	W	F	G	B	G
Information	F	W	G	B	F	B	G	B

*Step 10* Define the average values for the strategies.

Table 12 includes average values.

**Table 12:** Average values

	RLN				LDT				STY				TGL			
	PDG		NDG		PDG		NDG		PDG		NDG		PDG		NDG	
	μ	n	μ	n	μ	n	μ	n	μ	n	μ	n	μ	n	μ	n
Cost	.57	.35	− .57	− .35	.50	.31	− .55	− .34	.60	.37	− .45	− .28	.47	.29	− .45	− .28
Time	.48	.30	− .53	− .33	.50	.31	− .57	− .35	.57	.35	− .40	− .25	.57	.35	− .45	− .28
Volume	.58	.36	− .42	− .26	.55	.34	− .60	− .37	.48	.30	− .48	− .30	.60	.37	− .55	− .34
Information	.52	.32	− .57	− .35	.50	.31	− .45	− .28	.47	.29	− .45	− .28	.55	.34	− .43	− .27

*Step 11* Compute the score functions.

Table 13 calculates the score function values of the strategies.

**Table 13:** Score function values of the strategies

	RLN	LDT	STY	TGL
Cost	.278	.223	.235	.147
Time	.202	.234	.188	.209
Volume	.207	.292	.173	.292
Information	.244	.165	.147	.189

*Step 12* Normalize the decision matrix.

Table 14 gives information about the normalized matrix.

**Table 14:** Normalized matrix

	RLN	LDT	STY	TGL
Cost	.592	.478	.623	.341
Time	.430	.503	.499	.483
Volume	.440	.627	.458	.677
Information	.520	.354	.391	.438

*Step 13* Compute the weighted decision matrix.

Table 15 presents the weighted matrix.

**Table 15:** Weighted matrix

	RLN	LDT	STY	TGL
Cost	.111	.129	.194	.079
Time	.081	.136	.155	.111
Volume	.083	.170	.142	.156
Information	.098	.096	.121	.101

*Step 14* Determine concordance and discordance interval matrixes.

Concordance and discordance interval matrixes (CCM and DCM) are created in Table 16.

**Table 16:** CCM and DCM

Strategies	CCM				DCM			
	Cost	Time	Volume	Information	Cost	Time	Volume	Information
Cost	.000	.499	.499	.769	.000	.851	1.000	.311
Time	.501	.000	.311	.812	1.000	.000	1.000	.419
Volume	.501	.689	.000	.812	.662	.285	.000	.203
Information	.231	.188	.188	.000	1.000	1.000	1.000	.000

*Step 15* Compute aggregate index matrixes.

Aggregate index matrixes are demonstrated in Table 17.

**Table 17:** Aggregated index matrices

Strategies	CCM				DCM				Aggregated Matrix			
	Cost	Time	Volume	Information	Cost	Time	Volume	Information	Cost	Time	Volume	Information
Cost	0	0	0	1	1	0	0	1	0	0	0	1
Time	1	0	0	1	0	1	0	1	0	0	0	1
Volume	1	1	0	1	1	1	1	1	1	1	0	1
Information	0	0	0	0	0	0	0	1	0	0	0	0

*Step 16* Calculate net superior, inferior, and overall values for ranking the strategies.

Net superior, inferior, and overall values are computed in Table 18 for ranking the strategies.

**Table 18:** Net superior, inferior, and overall values of the strategies

Strategies	Net superior values	Net Inferior values	Overall values
Cost	.534	− .500	1.034
Time	.248	.284	− .036
Volume	1.005	− 1.850	2.855
Information	− 1.787	2.066	− 3.852

Ranking results are summarized in Table 19.

**Table 19:** Comparative overall ranking results for the risk-based strategic priorities of fintech lending

Strategies	Bipolar q-ROF multi SWARA-ELECTRE	Bipolar PF multi SWARA-ELECTRE	Bipolar IF multi SWARA-ELECTRE
Cost	2	2	2
Time	3	4	3
Volume	1	1	1
Information	4	3	4

**Fig. 5:**
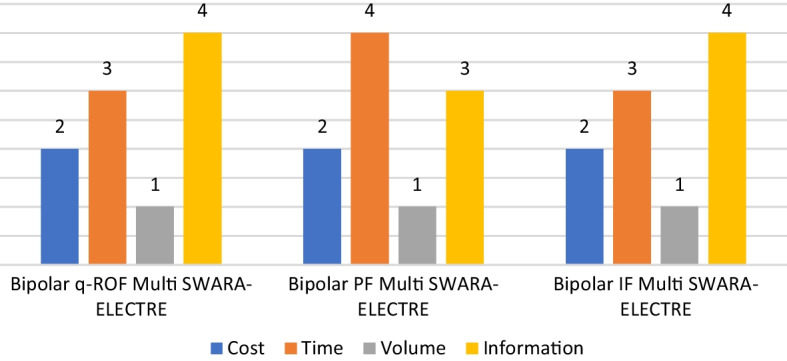
Graphical representations of the ranking results

The most important risk-based strategy for fintech lending is found to be volume. Secondly, cost should also be a top priority in this case. Time and data play the least important role. Figure 5 explains the ranking results in detail.

The results are validated using TOPSIS, and a sensitivity analysis is performed by altering the weights of the criteria in four separate cases. The comparative results are presented in Table 20.

**Table 20:** Validation and sensitivity analysis results

Strategies	Bipolar q-ROF Multi SWARA-ELECTRE	Bipolar PF Multi SWARA-ELECTRE	Bipolar IF Multi SWARA-ELECTRE	Bipolar q-ROF Multi SWARA-TOPSIS	Bipolar PF Multi SWARA- TOPSIS	Bipolar IF Multi SWARA- TOPSIS
*Case 1*						
Cost	2	2	2	2	2	2
Time	4	4	3	3	3	3
Volume	1	1	1	1	1	1
Information	3	3	4	4	4	4
*Case 2*						
Cost	2	2	2	2	2	2
Time	3	4	3	3	4	3
Volume	1	1	1	1	1	1
Information	4	3	4	4	3	4
*Case 3*						
Cost	2	2	2	2	2	2
Time	3	4	3	3	4	3
Volume	1	1	1	1	1	1
Information	4	3	4	4	3	4
*Case 4*						
Cost	2	2	2	2	2	2
Time	4	4	3	3	3	3
Volume	1	1	1	1	1	1
Information	3	3	4	4	4	4

In a number of instances, the comparative results of the extended M-SWARA and TOPSIS are remarkably similar. Consequently, it is possible to conclude that the proposed model is valid and that the weighting results are consistent with the shifting order.

## Discussions

Investors primarily take measures to mitigate the risk of unauthorized access to the IT systems of the fintech platform. The fintech lending system is conducted entirely online. Because all transactions on this platform are integrated over the internet network, there is a substantial IT risk associated with this system. Unavoidable IT risks will negatively impact the financing and functionality of fintech companies. These businesses must take the necessary cyber security precautions. Therefore, fintech companies will be accountable for any security issues that arise on this platform. This results in substantial financial losses for these companies. Meanwhile, fintech companies will suffer a significant loss of reputation due to this issue. Customers will not trust fintech companies with inefficient IT systems, thus threatening the continuity of fintech investments because it will result in customer defections.

In this context, fintech companies must take certain precautions to manage the security risk effectively. First, the primary threats to IT must be identified. In this context, all applications on the platform must be evaluated, and what risks exist and when they may manifest must. These risks must then be examined in depth. The probability of risk occurrence and their potential consequences should be determined during this step. Thus, it will be possible to rank the risks against one another. Next, control measures must be implemented to effectively manage these risks. These measures should be tailored to each type of risk. Finally, it must be determined if these measures effectively mitigate risks. In this context, security tests must be conducted for the implemented controls.

Numerous researchers have explained the significance of this situation in the literature. For instance, Iqbal et al. (2021), Ryu (2018), and Jagtiani and John (2018) concurred that fintech firms should take the necessary steps to effectively manage security risks. With the assistance of this issue, the risk management process can be conducted more efficiently, which positively impacts the companies' profitability. On the other hand, there are also studies in the literature with contradictory findings. For instance, Katsiampa et al. stated that the effectiveness of the fintech lending system should primarily take financial performance risks into account. In addition, Huibers (2021) highlighted the significance of regulation risk in this framework. It is determined that country-specific legal regulations should be considered in the design of this system.

It has been determined that increasing the number of financiers integrated into the system is the most effective strategy for boosting the success of the fintech lending system. This will allow for the ability to serve more customers. In this context, the fintech lending company must enter contracts with additional financiers. To convince these financiers, the platform must be safe, and the procedure must be profitable. Otherwise, investors will lack confidence in this platform and refuse to be integrated into the system. This will negatively impact the fintech system’s profitability. Similarly, Firmansyah and Anwar (2019), Acar and Çıtak (2019), and Sheikh et al. (2019) argued that reaching many financiers is essential for the effectiveness of fintech systems.

## Conclusions

This study evaluates significant risks and identifies the strategic priorities of fintech lending for clean energy projects. It developed a model to analyze a distinct set of risks and the strategic priorities of fintech lending for clean energy projects. M-SWARA methodology is used to analyze the risk factors of fintech lending. Furthermore, strategic priorities are evaluated using the ELECTRE method. These models are integrated with golden cut and bipolar q-ROFSs during this procedure. In addition to this issue, IFSs and PFSs are used for all calculations. Findings reveal that three additional risk factors affect security. Additionally, security influences the financial realm. Meanwhile, security is identified as the greatest risk factor for fintech lending systems, followed by financial. Financial and technological risks have lower significance. Volume is found as the most critical risk-based strategy for fintech lending. Moreover, cost should also be a top priority in this regard. Time and data play the least important role. Therefore, these companies must take the necessary cyber security precautions. In this framework, these companies can establish a new risk management department, which aims to identify the most significant threats to IT. The likelihood of occurrence and potential consequences of the risks should then be determined. Based on these findings, the necessary precautions should be taken to effectively manage these risks.

Constructing an impact-direction map of risk-based strategic priorities for fintech lending in clean energy projects and measuring the possible influences using a hybrid decision-making system with golden cut and bipolar q-ROFS are the most significant contributions to the literature. In this study, all risks associated with the fintech platform are included in the scope of the review. These risks could be addressed in a specific manner in a subsequent study. For instance, it is believed that a more comprehensive examination of IT risks would be advantageous. This application can be implemented for all types of risk. Additionally, the model developed for this study can be enhanced. In this context, the reliability of the model’s results can be tested by considering various fuzzy numbers.

## Data Availability

Not applicable.

## Abbreviations

ELECTRE: Elimination and choice translating reality
SWARA: The stepwise weight assessment ratio analysis
